# Activation of REG family proteins in colitis

**DOI:** 10.3109/00365521.2011.605463

**Published:** 2011-10-13

**Authors:** Atle Vand Beelen Granlund, Vidar Beisvag, Sverre H Torp, Arnar Flatberg, Per Martin Kleveland, Ann Elisabeth Østvik, Helge L Waldum, Arne K Sandvik

**Affiliations:** 1 Department of Cancer Research and Molecular Medicine, Norwegian University of Science and Technology, Trondheim, Norway; 2 Department of Laboratory Medicine, Norwegian University of Science and Technology, Trondheim, Norway; 3 Departments of Gastroenterology, St. Olav's University Hospital, Trondheim, Norway; 4 Departments of Pathology, St. Olav's University Hospital, Trondheim, Norway

**Keywords:** inflammatory bowel diseases, REGIA protein human, REGIV protein human

## Abstract

**Aims:**

To do a genome-wide gene expression study of active and inactive ulcerative colitis and Crohn's disease (inflammatory bowel disease – IBD) and examine the most differentially expressed genes. As the study showed an extreme upregulation of all regenerating islet-derived genes (REG proteins) in active IBD, we further studied the expression of REGs on protein level in active and inactive IBD, as well as in non-IBD (pseudomembranous) colitis.

**Methods:**

Microarray analysis was done on a total of 100 pinch biopsy samples from healthy controls and patients with Crohn's disease or ulcerative colitis. Tissue samples from IBD and pseudomembranous colitis were examined with routine histology and immunohistochemical analysis for REGIα, REGIV, DEFA6, and serotonin.

**Results:**

REG mRNAs were up to 83 times overexpressed in diseased mucosa compared with mucosa from healthy individuals. REGIα and REGIV were overexpressed at immunohistochemistry and located to different mucosal cell types. REGIα was expressed in basal half of crypts, REGIV in mid and outer parts of crypts and in surface epithelium and seems to be stored in, and secreted from, goblets. Pseudomembranous colitis samples showed similar staining patterns, and some IBD samples stained REG positive without inflammation on routine histology.

**Conclusions:**

All REG family mRNAs are upregulated in IBD. REGIα and REGIV have different cellular localization, possibly reflecting different biological functions. REG protein expression also in pseudomembranous colitis shows that REG family proteins are regulated in inflammatory injury and repair, not specifically for IBD as previously thought.

## Introduction

Regenerating gene (REG) family proteins are structurally similar proteins belonging to the calcium-dependent (C-type) lectin gene superfamily. In humans, the family has four known members; REGIα, REGIβ, REGIII, and REGIV. The first REG protein was discovered in regenerating pancreatic islets in rats [1], and REG proteins have since been found involved in other physiological and pathophysiological processes [2]. Their basic biological effects seem to be induction of cellular proliferation and inhibition of apoptosis. REGIα seems to have a physiological role in the gastric mucosa, related to the general trophic effect of gastrin on the gastric oxyntic mucosa [3]. Elsewhere in the gastrointestinal system, these proteins are found during tissue injury and neoplasia. They are overexpressed in gastric and colorectal cancers [4], and in colorectal cancer cell lines [5]. Lawrance et al. [6] first showed overexpression of *REGIα* and *REGIβ* mRNA in resected colonic tissue from both Crohn's disease (CD) and ulcerative colitis (UC). Subsequent studies have found that *REGIα, REGIβ* and *REGIII* mRNAs are overexpressed in the colon in inflammatory bowel disease (IBD) [7,8] and that *REGIα* is overexpressed in CD [9]. Sekikawa et al. found that *REGIα* mRNA and protein are overexpressed in UC [10], in particular in dysplasia or cancer, and a more recent study also shows a possible role for REG1α as a marker for UC associated neoplasia [11]. Very recent studies indicate a mechanism for IL-22 stimulated expression of REG1α through the IL-22R1 receptor [12].

In a microarray-based genome-wide gene expression study on CD and UC performed in our laboratory (unpublished), genes coding for REG family proteins and for Paneth-cell-specific defensins constituted six of the seven top genes on the list of differentially expressed genes in active CD. *REG* genes were up to 83 times overexpressed on mRNA level compared with tissue from healthy individuals. This finding prompted us to carry out a systematic study on the expression of all REG classes in affected and unaffected mucosa from patients with IBD. The expression of REGIα and REGIV was further investigated using immunohistochemistry (IHC). Since IBD specificity of REG gene induction is assumed but not proven, we also included an immunohistochemical analysis of REG proteins in pseudomembranous colitis (PC). This work thus describes the expression of *REGIα, REGIβ, REGIIIβ* and *REGIV* on mRNA level, and REGIα and REGIV proteins by IHC. Cellular localization of REGIα is studied by co-staining for the Paneth-cell-specific defensin alpha 6 (DEFA6), and of REGIV by co-staining with serotonin as a marker for enteroendocrine cells.

## Methods

### Clinical material

Patients admitted to the Gastrointestinal Endoscopy Unit, Department of Gastroenterology, St. Olav's University Hospital, for colonoscopy were included after informed consent. The patients had CD or ulcerative colitis/proctitis (UC/UP), or underwent colonoscopy due to gastrointestinal symptoms. The UC group also included six patients with an associated diagnosis of primary sclerosing cholangitis (PSC). Normal controls were defined by clinically indicated examinations finding no signs of gastrointestinal disease. Endoscopic biopsies were taken from macroscopically maximally inflamed mucosa, spanning the whole colon from rectum to ascending colon, and macroscopically normal tissue always from the hepatic flexure. Four adjacent biopsies taken from each location were either immediately snap frozen and stored on liquid nitrogen, or fixed on 4% buffered formaldehyde. For the IHC analysis, biopsies taken from patients with UP and antibiotic-induced PC were also included in the study. The Regional Medical Research Ethics Committee approved the study (no 5.2007.910), which was registered in the ClinicalTrials Protocol Registration System (identifier NCT00516776).

### Microarray analysis

The microarray analysis included a total of 100 samples, representing CD diseased/normal (7/19), UC diseased/normal (24/31), and normal controls (19). Frozen biopsies were homogenized and total RNA extracted using the Ambion *mir*Vana™ miRNA Isolation Kit (Applied Biosystems, CA, USA). RNA quality was determined using NanoDrop™ Spectrophotometer (Thermo Scientific, DE, USA) and Bio-analyzer (Agilent Technologies, CA, USA). All samples used were of high quality (Rin value >7). Microarray analysis followed standard protocols, using Illumina human HT-12 expression BeadChips (Illumina, San Diego, CA, USA) and an Illumina BeadStation.

The R software environment and the Bioconductor package were used for the analysis of the Illumina data [13]. Differential expression was assessed using linear models with least squares regression and empirical Bayes moderated t-statistics [14]. *p* Values were adjusted for multiple comparisons using Benjamini-Hochberg false discovery rate correction.

### Real-time RT-PCR

From the seven top differentially expressed genes (Table I), these six were verified by real-time RT-PCR: *REGIα, REGIβ, REGIIIβ, REGIV, DEFA5,* and *DEFA6.* Seven samples were randomly selected from each of five groups: CD (diseased and unaffected mucosa), UC (diseased and unaffected mucosa), and healthy individuals. Primer sequences (RefSeq Build 36.1) are given in Table II. The iScript cDNA synthesis kit (Bio-Rad, Hercules, CA, USA) was used to prepare cDNA, and real-time RT-PCR performed using a FastStart SYBR Green Master mix (Roche Diagnostics, Basel, Switzerland). Reference gene was β-actin (*ACTB*). PCRs were run in triplicate on an MX3000P™ System (Stratagene, La Jolla, CA, USA). The ΔΔ-Ct method was used [15] to analyze PCR data.

**Table I:** Microarray gene expression results – top 7 genes CDD/N.

Gene	CDD/N (ratio given as log_2_)	No – CDD	UCD/N (ratio given as log_2_)	No – UCD
*REGIα*	6,38	1	5,34	1
*REGIβ*	6,33	2	5,26	2
*REGIIIα*	6,14	3	4,58	4
*DEFA6*	5,45	4	2,89	29
*DEFA5*	5,29	5	2,80	30
*LCN2*	3,76	6	3,99	9
*REGIV*	3,73	7	3,48	13

UCD = diseased ulcerative colitis, CDD = diseased Crohn's disease, N = healthy controls. No – CDD = gene expression rank number on CDD prioritized list, No – UCD = gene expression rank number on UCD prioritized list.

**Table II:** Primer sequences for real-time RT-PCR verification of microarray results.

Gene	Sense primer (5’ – 3')	Antisense primer (5’ – 3')
*REGIα*	TCCATGACCCCAAAAAGAAC	TTAACAAGGCAAACTCAGCA
*REGIβ*	CACTGATGACAGCAATGTCT	GAAGGTACTGAAGATCAGCG
*REGIII*	GGAGTAGCAGTGATGTGATG	TAAAGCCTTAGGCCGTATGA
*REGIV*	CTCCTGGATGGTTTTACCAC	GATTCTTGCTCTATGGTCGG
*DEFA5*	GAAAGAGCTGATGAGGCTAC	TTGCACTGCTTTGGTTTCTA
*DEFA6*	GGATGCAAGCTCAAGTCTTA	TTGATGGCAATGTATGGGAC
*ACTB*	AAGATCATTGCCTCCTCCTGA	AATCTCATCTTGTTTCTGCG

Expression levels for unaffected and affected mucosa in UC or CD were compared with normal controls, using one-way ANOVA with Dunnett's post test. Statistical analyses were done and graphs generated using GraphPad Prism version 4.00 for Windows (GraphPad Software, San Diego, CA, USA).

### Histological and immunohistochemical examinations

Formaldehyde-fixed samples were embedded in paraffin, and 4-μm sections were cut and stained with hematoxylin-eosin (H-E) for histological evaluation by an experienced pathologist to assess inflammation. Biopsies used for immunohistochemical analysis were taken adjacent to those for gene expression analysis. Biopsies from PC were archival material; otherwise the biopsies were handled as for those in the IBD groups.

These commercially available antibodies were used: REGIα – polyclonal rabbit antibody (Cat No RD181078100, BioVendor, Midrice, Czech Republic, dilution 1:500), REGIV – polyclonal goat antibody (Cat No AF1379, R&D Systems, Minneapolis, MN, USA, dilution 1:400), DEFA6 – polyclonal rabbit antibody (Cat no HPA019462, Sigma, St. Louis, MO, dilution 1:1000), and serotonin – monoclonal mouse antibody (Cat no AB16007, Abeam plc, Cambridge, UK). REGIα DEFA6 and serotonin were detected with the Dako En Vision peroxidase kit (Dako, Glostrup, Denmark) and REGIV with the Vectastain ABC kit (Vector Laboratories, Burlingame, CA, USA), all combined with Dako DAB+ as chromogen. Sections were counterstained with hematoxylin. Serial (neighboring) sections were made to assess co-localization of REGIα and DEFA6, or REGIV and serotonin. A comprehensive study of REGIα protein expression was performed on a total of 147 samples, including samples from UC diseased/ normal (32/34) (including 4/5 patients with associated PSC diagnosis), CD diseased/normal (12/21), UP diseased/normal (11/13), and normal controls (23).

## Results

### Patients and biopsy specimens

Among the UC patients, 12 were using corticosteroids and 42 were using 5-aminosalicylic preparations, among the CD patients 6 and 5, respectively. All data were thoroughly assessed for correlation between medication and REG gene or protein expression. No such correlation was found. No patients used immunomodulants such as azathioprine or methotrexate, or TNFa blockers. Six patients were excluded due to a clinicopathological diagnosis of indeterminate colitis.

### Microarray and real-time RT-PCR analysis

Contrasting diseased CD or diseased UC against normal controls (CDD/N or UCD/N) generated lists of differentially expressed genes. Controlling the false discovery rate at 0.05, the number of significant genes was 4201 and 6935, respectively. The top genes when sorted according to gene expression ratio are shown in Table I. Gene expression levels were all significantly different from controls, adjusted *p* values ranging from 2.8 × 10^-10^ to 3.5 × 10^-6^. RT-PCR analysis confirmed the findings from microarray, mean gene expression ratio (log_2_) between diseased tissues versus normal controls varying from 3.8 for DEFA5 expression in UCD versus N to 11.7 for REGIβ expression in CDD versus N.

### Immunohistochemical analyses

**Figure 1:**
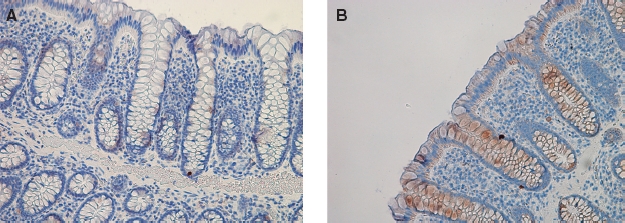
Representative samples with immunohistochemistry for REGIα (A) and REGIV (B) in control colonic biopsies from healthy individuals. REGIα staining is absent; REGIV shows slight staining in crypt and superficial mucosal cells.

**Figure 2:**
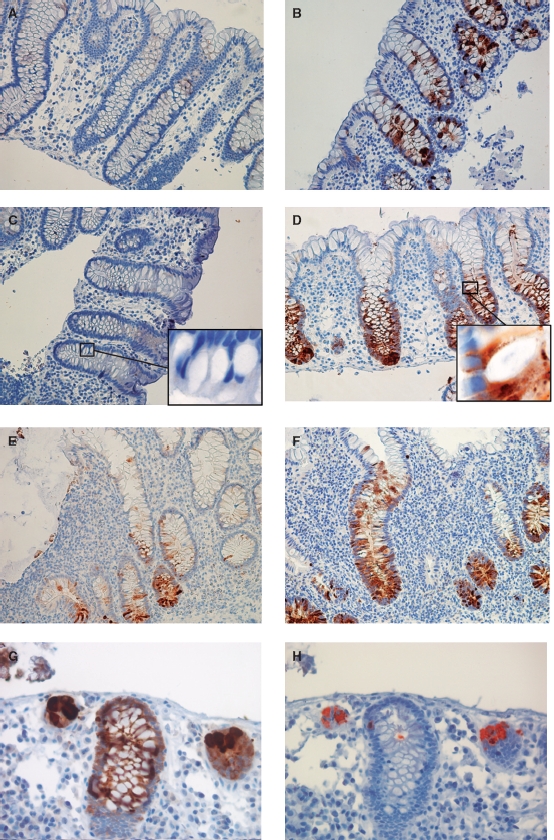
(A)/(B): Immunohistochemistry (IHC) for REGIα in non-inflamed mucosa from patients with known Crohn's disease (CD) (panel A, negative immunostaining; B, positive). (C)/(D): REGIα in non-inflamed mucosa from patients with ulcerative colitis (UC) (panel C, negative immunostaining; D, positive). Insert C shows negative staining in non-inflamed mucosa and insert D shows REGIα in cytoplasma of goblet cells but no staining inside gobletseF: Positive IHC for REGIα in inflamed mucosa from patients with CD (E) and UC (F). (G)/(H): Basal colonic crypts from non-diseased mucosa in ulcerative colitis stained for REGIα (G) or DEFA6 (H). Crypt epithelial cells show a general cytoplasmic staining for REGIα, and REGIα staining is clearly stronger in the DEFA6-positive Paneth cells.

**Figure 3:**
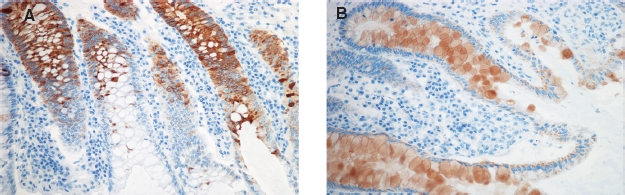
Immunohistochemistry for REGIα (A) and REGIV (B) in mucosa from patients with known pseudomembranous colitis. Positive immunostaining is shown for both antibodies, in a pattern similar to the one observed in IBD samples (Figures 3 and 4).

Normal colonic mucosa had no REGIα immunore-activity (Figure 1A). In patients with CD and UC biopsies from mucosa histologically assessed as normal, immunoreactivity for REGIα ranged from negative to moderate positively stained crypt epithelium (Figure 2A-D). The staining pattern for PC samples (Figure 3A) was similar. Diseased mucosa from all patient groups revealed strong REGIα immunostaining in cytoplasm of crypt cells whereas surface epithelium was negative (Figure 2D (insert)). DEFA6 immunoreactive epithelial cells were localized in the basal parts of the crypts. REGIα was expressed in both Paneth cells and crypt epithelium, in Paneth cells at a higher level (Figure 2G-H). The results from REGIα immunostaining of the extended set of samples are shown in Table III.

**Table III:** Summary of results from immunohistochemical evaluation of REGIα levels in biopsies.

		IHC evaluated REGIα level						
				
Sample Group	Number of samples	P	0	1	2	3	Mean REGIα level	Differnt from normal group (*p* value)
N	23	1	22	0	0	0	0.0	–
CDN	21	5	14	0	1	1	0.4	NS
UCN	34	4	27	1	1	1	0.2	NS
UPN	13	3	10	0	0	0	0.1	NS
CDD	12	1	3	1	5	2	1.5	<0.01
UCD	32	2	5	5	13	8	1.7	<0.01
UPD	11	0	2	4	3	2	1.4	<0.01

N = healthy controls; CDN, UCN, or UPN = histologically normal mucosa from patients with known Crohn's disease, ulcerative colitis, or ulcerative proctitis; CDD, UCD, or UPD – histologically diseased mucosa from patients with Crohn's disease, ulcerative colitis, or ulcerative proctitis.

REGIα expression level: “P": only Paneth cells were stained, “0” to “3": an increasing level of staining. 0: No staining; 1: <20% of crypt epithelial cells stained; 2: 20-50% of crypt epithelial cells stained; 3: >50% of crypt epithelial cells stained. Difference from normal group is evaluated using two-tailed Welch t-test and result given as the *p* value for this test.

**Figure 4:**
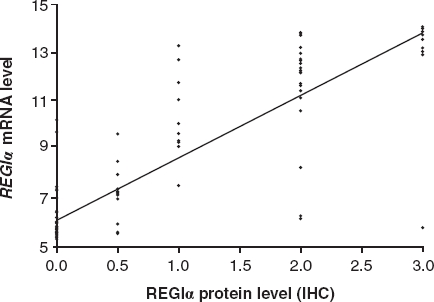
Correlation plot of *REGIα* mRNA levels measured using microarray and REGIα staining levels observed using immunohistochemistry (IHC; as given in Table III). Correlation analysis showed a strong correlation with squared correlation coefficient (R^2^) of 0.76 and *p* value < 0.0001.

A correlation analysis investigating the relationship between *REGIα* gene expression detected in micro-array analysis and REGIα protein expression observed during IHC showed a significant correlation, with a squared correlation coefficient (R^2^) of 0.76 and *p* value < 0.0001. A plot of the correlation with the regression line fitted is shown in Figure 4.

**Figure 5:**
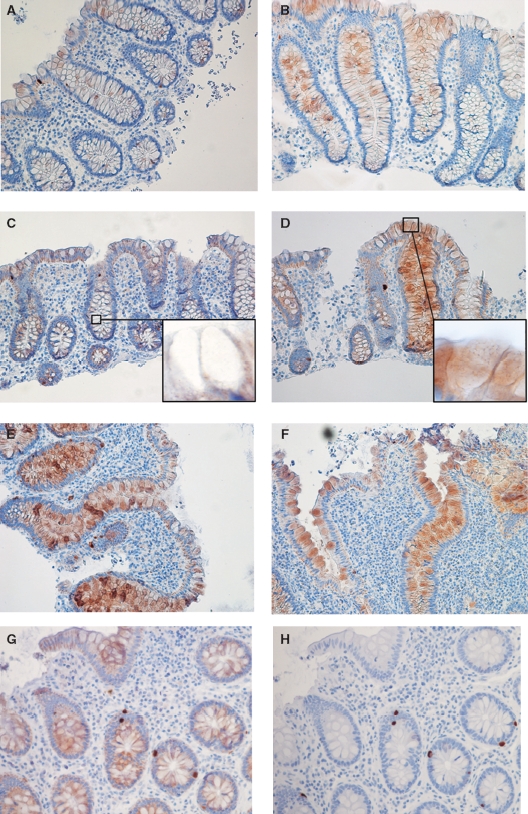
(A)/(B): Immunohistochemistry (IHC) for REGIV in non-inflamed mucosa from patients with known Crohn's disease (CD). (C)/ (D): REGIV in non-inflamed mucosa from patients with ulcerative colitis (UC) (panel C, negative immunostaining; D, positive). Insert C shows empty goblets in non-inflamed mucosa and insert D shows REGIV-filled goblets and REGIV-positive material being extruded into the lumen. (E)/(F): Positive IHC for REGIV in inflamed mucosa from patients with CD (E) and UC (F). (G)/(H): Basal colonic crypts from non-diseased mucosa in Crohn's disease stained for REGIV (G) or serotonin (H), showing that serotonin-positive enteroendocrine cells are also positive for REGIV.

Slight REGIV immunoreactivity was observed in crypts and surface epithelium of all control biopsies (Figure 1B). Biopsies from IBD cases assessed as normal revealed negative to weakly positive immunoreactivity (Figure 5A-D), often exceeding that of controls. Inflamed mucosal biopsies from both IBD (Figure 5E and F) and PC (Figure 3B) showed moderate to strong REGIV immunostaining with similar histological distribution. In goblet cells, immunoreactivity was localized to the mucin contents and excreted material (Figure 5D (insert)). Additionally, REGIV immunoreactivity was found in both healthy and diseased individuals in cells that co-stained for serotonin (Figure 5G-H), indicating that these were enterochromaffin cells. This co-staining pattern was similar in healthy individuals and in normal and inflamed mucosa of IBD and PC patients.

## Discussion

This study shows a massive overexpression of *REG* family mRNAs in diseased colonic mucosa with CD or UC. As there are no commercially available antibodies for REGIβ and REGIIIα, only REGIα and REGIV were also studied by IHC. For these two REGs, the overexpression of *REG* mRNAs was confirmed on protein level, with strong staining for REGIα and REGIV in all cases of active IBD. Correlation analysis showed a strong correlation between microarray measurement and IHC evaluation of REGIα, indicating that the mRNA levels indeed reflect protein levels. Samples from healthy individuals were negative for REGIα, but had a slight background staining for REGIV. A corresponding overexpression on protein level was seen in some samples from patients with known IBD but no inflammation on routine histology and in non-IBD colitis samples. These findings have not been reported previously.

The cellular localization of REGIα and REGIV is different. REGIα positivity is seen in basal crypt cells, REGIV mainly in the mid-gland areas. REGIα was located to the cytoplasm, increasing during inflammation. Cytoplasmic REGIV was not clearly different between biopsies from healthy individuals, active IBD or inactive IBD. However, REGIV was clearly increased in goblets in active disease and seemed to be secreted with the mucus. The observation that REGIV is located to goblets corresponds to previous findings by Kamarainen and coworkers [16], and a recent study on appendiceal mucinous cystadenoma and pseudomyxoma peritonei [17] also found REGIV in goblets and mucus. This suggests that REGIV is secreted and may have a luminal mode of action. Additionally, REGIV is clearly localized to serotonin-positive enteroendocrine cells of the colon as has previously been observed in the upper GI tract and in the ileum [16]. This is of potential interest since serotonin seems to have an unclarified role in IBD [18]. At least one study suggests that REGIα is found solely in Paneth cells [8]. In our study, REGIα is found in both Paneth and goblet cells and Paneth cells staining stronger than other crypt cells. Thus, both Paneth and goblet cells express REGIα but at a higher level in Paneth cells. Interestingly, REGIα staining is seen in some cases with CD or UC, where routine histological examination concluded with normal colonic mucosa. The positivity was mainly seen in Paneth cells, but in a few cases also in other cells of the epithelium (see Table III for details). This suggests that REGIα IHC could aid in the diagnosis of IBD when biopsies are taken in the quiescent phase of the diseases.

REG proteins are antiapoptotic and stimulate proliferation and repair [2], and apparently have roles in the trophic response to gastrin and in colorectal cancer. This indicates that REG proteins are involved in injury, repair, and growth on a general level. This assumption is supported by a REGIα expression pattern in pseudomembranous colitis indistinguishable from that in IBD samples. It is possible that REGIV has functions distinct from other REG proteins. It is less regulated than any other *REG* mRNA species in IBD; it is localized to another population of epithelial cells and is also found in enteroendocrine cells. Contrary to REGIα this protein seems to be constitutively expressed, at least in serotonin-positive enteroendocrine cells. Another highly interesting aspect of REG proteins in the gut is that Reglllγ in the mouse and its human ortholog REGIIIα are considered to be antimicrobial peptides with a lectin-like mode of action [19]. There are no reports of this function for other REG proteins, but with REGIV seemingly being excreted to the lumen, further studies on the antimicrobial effects of REG proteins are certainly warranted.

This is the first study with a comprehensive evaluation of REG proteins in IBD, which also includes non-diseased IBD samples and a non-IBD inflammatory disease of the colon. We conclude that REG family proteins are strongly regulated in IBD and most likely involved in injury and repair. At least one protein in the family, REGIV, may have a topical mode of action and be involved in the enteroendocrine aspect of IBD pathophysiology. The REG protein response is not specific for IBD, but can be found also in pseudomembranous colitis. Finally, REGIα is a hitherto unrecognized and possibly useful and sensitive marker for mucosal injury and repair.
